# The Curious History of L'Hotel-Dieu De Paris

**Published:** 1913-03-29

**Authors:** 


### THE CURIOUS HISTORY OF L'HOTEL-DIEU DE PARIS.*

### II.—THE ACCOMMODATION FOR PATIENTS.

The hospital was not originally founded specially for the care of the sick, but as an asylum open to all in need of succour. The indigent, the aged, the infirm, the traveller, and the pilgrim, as well as the sick man, found shelter within its walls. There existed in the Hôtel-Dieu, at any rate in the Middle Ages, another class of inmate, called pensioners or boarders.

### Paying Patients and Life Annuitants.

On paying an annual fee, handing over goods or promising to do so after death, it was possible to obtain quarters in the building after signing a contract for a fixed time or for life. Little by little, however, the sick began to predominate, and from the sixteenth century onwards the hospital was practically reserved for their use. All persons except lepers were entitled to admission, without distinctions of age, sex, or race in times of sickness, or when pregnant. At first the number of beds available was low, but in the twelfth century it increased rapidly owing to the Capitular Chart of 1188, which obliged every Canon of Notre Dame who renounced his prebendaryship or was on the point of death to leave a bed—that is to say, a mattress, pillow, and sheets—to the Hôtel-Dieu. By the fifteenth century the number of beds had reached 303, and by the following century 500. The maximum was arrived at in the eighteenth century—namely, 1,219; but the new hospital only contained 829. Up to the time of the Renaissance the Hôtel-Dieu was the only hospital in Paris, and, consequently, its work was much hampered in times of stress, due to famine, civil war, and epidemics. It was the plague in the sixteenth century which led to such overcrowding that a second hospital, La Charité, became necessary. This was started in 1519, and a third, Saint-Louis, in 1607. In the eighteenth century, during the famine and the accompanying epidemic of scurvy, no less than six thousand sick were accommodated in the Hôtel-Dieu alone.

It was not uncommon in the fifteenth and sixteenth centuries for any number of patients up to twelve to be placed in a single bed, and later the number is said to have risen to fourteen. As may be supposed, the beds were large, and capable of holding normally—if one may use the word—three persons. In the eighteenth century two types of bed were in use—large ones meant to hold four and small ones to hold one person. Of the 1,219 beds then possessed by the hospital, 733 were of the larger kind. It is, however, rather startling to be told that fourteen persons were placed in a bed meant to hold four, even though the accommodation were relatively increased by placing several beds side by side! Up to the seventeenth century four-poster beds were in common use, and it was then that the idea struck Geneviève Bouquet of accommodating a number of the more convalescent patients on the solid canopies of these beds. This was during the war of La Fronde, when the hospital was greatly overcrowded. The patients had to make use of ladders to reach their perches. In spite of the increased accommodation thus ensured and made use of for many years, it was found necessary during the terrible famine that occurred in the reign of Louis XIV. to admit no fewer than eight patients into the "interior" of the beds. It is not difficult to imagine the consequences of such herding in times of pestilence. The death-rate then assumed enormous proportions. It is stated that in 1592 no fewer than 63,000 persons died of plague in the Hôtel-Dieu alone. In the Middle Ages candidates for admission were examined by the portress, a nun. In the eighteenth century and up to the time when *internes* or house surgeons were appointed, this duty devolved upon the *compagnon chirurgien visiteur*. After admission came the obligatory confession, then the disrobing, next the putting to bed, and, lastly, the sending of the clothes to the *pouillerie*, where they were cleansed and stored. It was only in the thirteenth century that "men of art" penetrated into the hospital. In 1221 the surgeon Hubert, "out of charity and for the good of his soul," took it upon himself to visit the sick of the Hôtel-Dieu.

### Medical Services and Midwifery.

About the same time Vincent des Bois gave his services in addition to a large sum of money to the hospital. After 1328 the patients were treated by two surgeons nominated by the King and paid for out of public funds. The services of a physician and also of a midwife called the *ventrière des accouchées* were also engaged. Things continued in this way for 200 years until the medical staff came under the administration of, and was regulated and paid by, the hospital itself. At that time only one physician, Mathurin Thabouet, was on the staff, and he was not obliged to pay more than one or two visits each week. The growing activity of surgery, however, necessitated a much larger staff, consisting of a master-surgeon, a surgeon-companion, about to be given his mastership, surgeon-companions for work in the wards, and surgeon-apprentices for work in what corresponded to the out-patient department, and in private. "Bleedings," of which more than 400 took place daily, provided work for all these without taking other operations into consideration. Thus, as it pleases Ambroise Paré, who lived there from 1530 to 1536 in the capacity of surgeon-companion, to state, extensive experience could be gained at the Hôtel-Dieu. In 1633 the hospital had three physicians on its staff, and by 1787 the number had increased to eleven, among whom was Fagon the celebrated physician of Louis XIV. The Revolution abolished the name of the Deity from the calendar, and the institution was called "Grand Hospice de L'Humanite." In 1829 nomination was superseded by competitive examination and the invidious distinction abolished. This method of obtaining appointments is the one in vogue to-day.

* *Continued from page* 626.

